# Low molecular-weight gel fraction of *Aloe vera* exhibits gastroprotection by inducing matrix metalloproteinase-9 inhibitory activity in alcohol-induced acute gastric lesion tissues

**DOI:** 10.1080/13880209.2017.1371770

**Published:** 2017-09-05

**Authors:** Chul-Hong Park, Hyeong-U Son, Chi-Yeol Yoo, Sang-Han Lee

**Affiliations:** a Department of Food Science and Biotechnology, Graduate School, Kyungpook National University, Daegu, Korea;; b Radiation Research Division for Biotechnology, Advanced Radiation Technology Institute, Korea Atomic Energy Research Institute, Jeongeup, Korea;; c Food and Bio-Industry Research Institute, Kyungpook National University, Daegu, Korea

**Keywords:** Antiulcer activity, alcohol induction, ulcer index, RT-PCR, immunohistochemistry

## Abstract

**Context:***Aloe* has been used for the prevention and cure of various diseases and symptoms including burns, injuries, oedema and pain.

**Objective:** This study determines the specific inhibitory activity of matrix metalloproteinase (MMP)-9 induced by the low molecular-weight gel fraction of *Aloe vera* (L.) Burm.f. (lgfAv) on alcohol-induced acute gastric lesions.

**Materials and methods:** We examined the protective effects of oral (p.o.) administration of lgfAv (molecular weight cutoff <50.0 kDa, 150.0 mg/kg body weight) in a Balb/c mouse model of alcohol-induced acute gastritis for 1 h exposure. By measuring ulcer index, we compared the antiulcerative activity of the fraction. mRNA expression and immunohistochemical analysis of various biomarkers were performed.

**Results:** The lgfAv-treated mice exhibited drastically fewer ulcer lesions than the untreated control mice did. It featured that lgfAv lessened the ulcer lesions than their relevant controls. Moreover, the transcriptional level of MMP-9 was completely alleviated by lgfAv treatment in alcohol-treated gastritis-induced mice.

**Discussion:** The transcriptional level of MMP-9 was significantly alleviated by lgfAv treatment of the model. However, reverse transcription-polymerase chain reaction (RT-PCR) and immunohistochemistry experiments revealed that lgfAv treatment in mucosal tissues had the potential to inhibit the mRNA and protein expression levels of MMP-9, respectively. The protein expression of MMP-9 was closely associated with lgfAv-induced gastroprotection against alcohol-induced gastric lesions.

**Conclusions:** The present findings suggest that lgfAv has the potential to alleviate alcohol-induced acute gastric lesions, which is mediated in part, mainly by the suppression of the mRNA expression of MMP-9.

## Introduction

*Aloe vera* (L.) Burm.f. (Av) is a worldwide plant that grows easily in varying climates, and is now widespread in Africa, America, Asia, Europe and other areas. Av has been extensively investigated to improve its use as an anti-inflammatory or wound healing agent (Pazyar et al. 2014). Av extract is believed to promote the wound healing of scorches, cutaneous injuries, oedema, and effectively reduce pain (Izzo et al. 2016). The gel has been exhibited to prevent gastric ulceration. The extract also has potential in promoting anti-inflammatory, antidiabetic, cell protective, healing and mucus stimulatory activities (Izzo et al. 2016). The numerous activities and underlying molecular mechanisms of the fractions and components of Av have not been fully documented, although the molecular mechanisms have been proven to exhibit wound healing and inflammation mitigation.

Furthermore, ulcerative mucosal lesions have created a complex clinical problem worldwide, because their presence evokes severe gastric ulcer or related symptoms (Satoh and Takeuchi 2016). To accelerate the research and development of cures for acute and chronic gastritis, researchers have developed some gastric ulceration models such as water immersion-restraint stress (WIRS), alcohol, lipopolysaccharide (LPS) or indomethacin in laboratory animals (Cho and Ogle 1992). Those models have been utilized to observe the numerous factors affecting the healing of gastric injury (Evangelista 2006). Alcohol-induced gastric ulceration has been used to investigate the mechanisms of acetaminophen, *N*-acetyl cysteine and prostaglandins, whereas the WIRS model has been applied to investigate the mode of action of verapamil, *N*-ethylmaleimide and cimetidine during ulcerogenesis (Maity et al. 2003). This implies that the two types of gastric injury may allow other ulcerogenic mechanisms (Maity et al. 2003). Under normal conditions, gastric microenvironment is controlled by signalling mediators such as matrix metalloproteinases (MMPs), prostaglandins and cytokines, which take crucial part in maintaining gastromucosal homeostasis as well as in regulating acute gastric lesions or the healing process (Hatazawa et al. 2006).

Previously, we reported that the polymer fraction (a mixture with molecular weight >50 kDa) exhibited protective activity in an alcohol-induced gastric lesion mouse model (Park Heo et al. 2011; Park, Son et al. 2011). Therefore, here, we evaluated the potential gastroprotective effects of the low-molecular weight fraction (with molecular weight cutoff, MWCO <50 kDa) in ameliorating gastric mucosal damage in mice, and investigated if these effects were mediated by regulation of the mRNA expression of MMP-9. Finally, our data suggest that MMP-9 expression may be a robust and reproducible biomarker of alcohol-induced acute gastric lesions.

## Materials and methods

### Reagents

The RNA extraction was performed using TRIZOL^®^ reagent (Invitrogen, Carlsbad, CA). The alcohol (absolute alcohol, 99.8%, Sigma-Aldrich Corp., St. Louis, MO), QA-Agarose (Qbiogene, Inc., Irvine, CA), AMV-RTase and *Taq*-polymerase (Solgent, Daejeon, Korea) were commercially available. The omeprazole (5-methoxy-2-[{(4-methoxy-3,5-dimethyl-2-pyridinyl)methyl} sulfonyl]-1H-benzimidazole, mw = 345.42) was obtained from Sigma-Aldrich Corp. (St. Louis, MO), and was used as the positive control. Other reagents were commercially available.

### Preparation of low molecular-weight gel fraction

The low molecular-weight gel fraction of Av (lgfAv) was obtained as described previously (Baek et al. 2010). In brief, we ultrafiltered the supernatant (335 kg) of the fresh *Aloe* gel, and then fractionated the filtrate using a laboratory scale UF system (Tami Industries S.A., Nyons, France). During the purification process, the transmembrane pressure (TMP) drop was operated at 0.2–2.25 bar under 23 °C at a speed of 240 L/h, respectively. We separated the filtrates of the 50 kDa MWCO fraction and finally collected the permeate fraction (158.6 kg) by removing the retentate fraction (149.2 kg). The precipitates were freeze-dried (0.5 Torr for 24 h), and pulverized using an 80-mesh filter to obtain the gel fraction (∼632 g, 0.094% yield). The voucher specimen of the fraction was deposited in Lab of Food Enzyme Biotechnology, Kyungpook National University, Daegu, Korea.

### Measurement of acid neutralizing capacity

To measure the potential acid neutralizing capacity of the fraction, we first prepared the test sample by mixing 100 mg of the sample and 3 mL distilled water (DW) with glass beads in a 5 mL tube (Panda and Shinde 2016). After incubating this mixture overnight with shaking at 23 °C, it was filtered after adding 0.1 N hydrochloric acid (HCl), followed by incubation with shaking for 1 h at RT, and then 20 μL 0.1% methyl orange was added for further titration using 0.1 N sodium hydroxide (NaOH).

### Animals and husbandry

The six-week-old specific pathogen-free (SPF) male BALB/c mice (28 mice; average weight, 20–23 g) were obtained from Samtaco Korea (Osan, Korea). They were fed a commercial diet (Samtaco, Osan, Korea) and provided water *ad libitum*. The animal care was accomplished according to the in-house Guidelines of Kyungpook National University Animal Care and Use Committee (KNU-2014-0114), and the current protocols were executed in accordance with the Guidelines of the International Association for the Study of Pain Committee for Research and Ethical Issues (Charlton 1995). All animals were adapted to the laboratory environment for at least 1 week prior to the animal experiments.

### Alcohol-induced acute gastric lesions and measurement of ulcer index

The alcohol-induced acute gastric ulceration model was established as described elsewhere (Bucciarelli et al. 2010). The mice were randomly divided into four groups: control (no treatment), alcohol (8 mL/kg), omeprazole (as a positive control agent, 3 mg/kg) and lgfAv (150 mg/kg) with alcohol treatment (*n* = 7 mice/group). Omeprazole was used to compare the protective effects of the fraction against acute gastric lesions (Bucciarelli et al. 2010). Before administration, the animals were fasted for 24 h, but allowed to tap water *ad libitum*. The omeprazole-treated group received one oral (p.o.) dose (3 mg/kg) dissolved in saline, the lgfAv-treated group were administered 150 mg/kg, and the mice in the control group received one p.o. dose of saline. After administration of the test material, the mice were returned to their cages for 30 min to allow absorption, and then they were administered (p.o.) alcohol (8 mL/kg). After exposure to alcohol for 1 h, the mice were euthanized for anatomical observations. The gastric lesions were measured by scoring each point according to the severity of the haemorrhagic erosions, as reported previously (Park Heo et al. 2011; Park, Son et al. 2011).

### Image analysis of gastric lesion tissues

**Figure 1.**
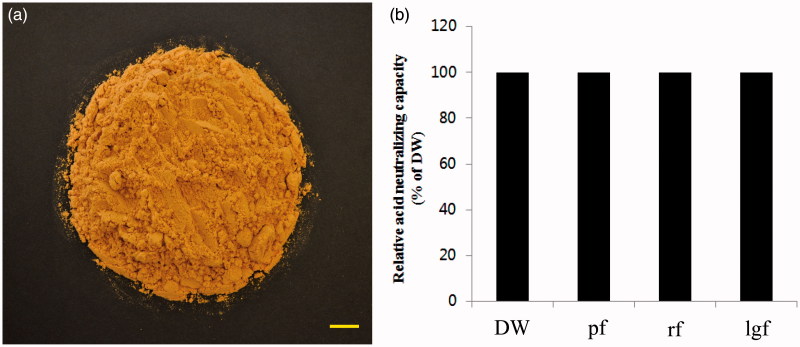
Classical feature of *Aloe vera* gel fraction and measurement of acid neutralizing potential. Scale bar, 1 cm. DW: distilled water; pf: polymer fraction; rf: retentate fraction; lgf: low molecular-weight gel fraction. Details of preparation method of fractions are included in Materials and Methods section (a). Relative acid neutralizing capacity was determined (b).

To measure the ulcer index score using image analysis, we examined specific features such as stripe erythema (or dot-shaped lesions) and various types of damage to the inner stomach tissues. Therefore, we subdivided the score level according to the degree of damage, which was classified a slight or severe. To ensure the images acquired were consistent, the light source and digital raw file were controlled (Figure 1(b)). To avoid the effect of sunlight and interior lighting, the photography was carried out in a darkroom using a strobo (Hyundai Fomax E400, Seoul, Korea). The camera used was a Nikon digital camera D5100 (Nikon Co., Tokyo, Japan) with an AF-S Micro NIKKOR 40 mm f/2.8 G lens (Nikon Co., Tokyo, Japan) and a Nikon ML-L3 wireless remote control. Initially, a colour image scale (QP card 203, QPcard AB, Helsingborg, Sweden) was photographed after the picture target was placed in the mini studio, which consisted of a diffuser and background fabric cover. The colour image scale is a useful tool for controlling the colour profile and enables the novel reproducibility of colour character and colour temperature (Lee et al. 2015). The gastric organ images were also captured using the same colour image scale data conditions in the mini studio. The photographs of the gastric organs were saved as a raw file, and the colour was adjusted using the QP calibration v1.21 software (QPcard AB, Helsingborg, Sweden).

### mRNA expression

The frozen gastric tissues (approximately 100 mg) were mixed in 1 mL of TRIZOL^®^ reagent, and the total RNA was arranged as described previously (Lee et al. 2015). The total RNA content was analysed using a multilabel counter (VICTOR3 spectrophotometer, Perkin Elmer, Turku, Finland) at 260 nm, and the RNA purity was determined using 1% formaldehyde-agarose gel electrophoresis and ethidium bromide staining. The RNA samples were then preserved at −80 °C until use. The reverse transcription was carried out using the avian myeloblastosis virus (AMV)-reverse transcriptase (RTase), and the polymerase chain reaction (PCR) was performed using the *Taq*-polymerase and the following specific primer sets: epidermal growth factor (EGF), forward (F): 5′-ACTCGGAAGCAGCTATCAAACC-3′ and reverse (R): 5′-CCTCTATTTCCTGGGGTCCTCT-3′, 337 bp; β-cellulin (BTC), F: 5′-CTGGTGGTCTGCTTGATAGTGG-3′ and R: 5′-CCTGAGACATGTCCTGTCCAT C-3′, 256 bp; cyclooxygenase (COX)-1, F: 5′-ATCCCATCTGTTCCCCAGAGTA-3′ and R: 5′-ACGAAAACCCACATCAAGGACT-3′, 267 bp; COX-2, F: 5′-TCGATGTCATGGAACTGTACCC-3′ and R: 5′-TAGGCTGTGGATCTTGCACATT-3′, 270 bp; iNOS, F: 5′-AACCCAAGGTCTACGTTCAGGA-3′ and R: 5′-TTACTCAGTGCCAGAAGCTGGA-3′, 335 bp; nNOS, F: 5′-AAGAACAAGGGCGTCTTCAGAG-3′ and R: 5′-TAAGGCGGTGGTCACTTCATA-3′, 328 bp; eNOS, F: 5′-TGCACAGGAAATGTTCACCT-3′ and R: 5′-GAGTAACAGGGGCAGCACAT-3′, 271 bp; mucin 5AC (MUC5AC), F: 5′-CCATGTGTATTCCTCTCCCACA-3′ and R: 5′-CTGACCCAGATCCTCCATCTCT-3′, 304 bp; and 18 S RNA, F: 5′-ATGTGGTGTTGAGGAAAGCAG A-3′ and R: 5′-TCTTGGATACACCCA CAG TTC G-3′, 327 bp. After the reaction process (18–34 cycles of 30 s at 95 °C, 30 s at 56 °C and 45 s at 72 °C), each product was analysed using 1% of agarose gel electrophoresis imaged using EtBr staining. The gene product was confirmed by comparison to a 1 kb DNA ladder (Solgent, Daejeon, Korea). For the semi-quantitative analysis, the relative intensity of the gene products in each set was standardized using the Molecular Imager (ChemiDoc XRS^+^, Bio-Rad, Philadelphia, PA).

### Immunohistochemical analysis

The immunohistochemical analysis was achieved as described previously (Rohani and Parks 2015). Briefly, the tissues were embedded in paraffin, cut into 5 mm sections, and set on a slide warmer for 16 h. After deparaffinization with xylene, the sections were subjected to serial dilutions of alcohol from 70 to 100% for 60 min each. After washing, the tissue sections were immunohistochemically analysed by first incubating them with 10% normal goat serum for 1 h at RT to block the nonspecific binding. Then, the sections were developed at 4 °C for 16 h with a rabbit anti-MMP-9 antibody (1:200, Santa Cruz Biotechnology, Dallas, TX).

### Data analysis

Data are presented as means ± standard deviation (SD). The statistical values were analysed using an ANOVA with the Tukey *post hoc* test (Park, Son 2011). *p* Values <0.05 were set as statistically significant.

## Results

### LgfAv showed no acid neutralizing capacity

We first prepared various Av fractions to evaluate their anti-inflammatory activity. We obtained the lgfAv as a pure fraction using a laboratory-scale ultrafiltration system (0.094% yield). The fraction was prepared using a freeze-drying method and finally obtained as a powder (Figure 1(a)). The acid neutralizing potential of the powder sample was subsequently determined. As shown in Figure 1(b), the lgfAv fraction did not show any acid neutralizing activity.

### Effects of lgfAv on mucosal tissues of alcohol-induced gastric lesion model

**Figure 2.**
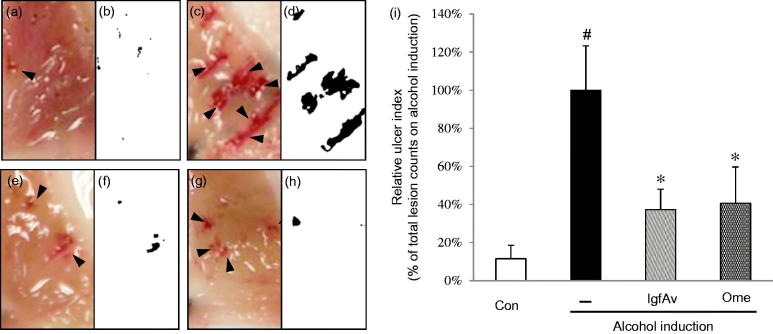
Gastroprotective effects of *Aloe vera* gel fraction on mucosal tissues in alcohol-induced acute gastric lesion model. Mice were randomly divided into (a and b) control, (c and d) alcohol alone (8 mL/kg), (e and f) gel (150 mg/kg) and (g and h) omeprazole (3 mg/kg) with alcohol treatment (8 mL/kg; *n* = 7). After oral (p.o.) administration, mice were returned to their cages for 30 min to apply drug metabolism. Mice were not allowed food or water during alcohol induction. Mice were euthanized, and the tissues were anatomically examined for visible signs such as scars or clumps of blood caused by ulcer, or both. (i) Gastric lesion index was measured and expressed relative to intensity of control. Gastric lesions were counted as described in Materials and methods section. Data are mean ± standard deviation (SD, *n* = 7 animals/group). #*p* < 0.05 and *p < 0.05 compared with control and alcohol-administered group, respectively. Results were analysed using multiple range Dunnett′s test. Con: control; –: alcohol induction alone; lgfAv: low molecular-weight fraction of *Aloe vera*; Ome: omeprazole. Each image (a–h) is divided into two parts: clinical observation and image analysis (see Materials and Methods for details).

As you can see Figure 2(a,b), the alcohol-induced groups showed severe gastric lesions of 1–2 mm in size (compare Figure 2(a,c)). In contrast, the positive control mice expressed very few lesions whereas those of the lgfAv-treated mice were significantly mitigated (Figure 2(e)) and only small-sized gastric lesions were detected (compare Figure 2(e,g), as the positive control). Therefore, the current results indicate that lgfAv-treated mice exhibited few lesions; moreover, their sizes were smaller than those of the alcohol-induced and omeprazole-treated mice were. We measured the ulcer index by scoring the lesions, as shown in Figure 2(i). The lgfAv-treated group exhibited a dramatically reduced ulcer index of 62%, which was better than that of the omeprazole-treated group (60%) and compared with the alcohol-treated group, which was considered as 100% index (compare Figure 2(e,c,g)). Furthermore, by capturing images of the specific damage in tissues, we also confirmed whether the tissues were healthy or not. In addition, we compared two methods: gastric lesion index measurement and image analysis, and the results showed the same pattern for the measurement of the lesions (compare images in Figure 2d, f and h). These results indicate that the lgfAv fraction alleviated the alcohol-induced gastric lesions *in vivo*.

### Immunohistochemical and RT-PCR analyses

**Figure 3.**
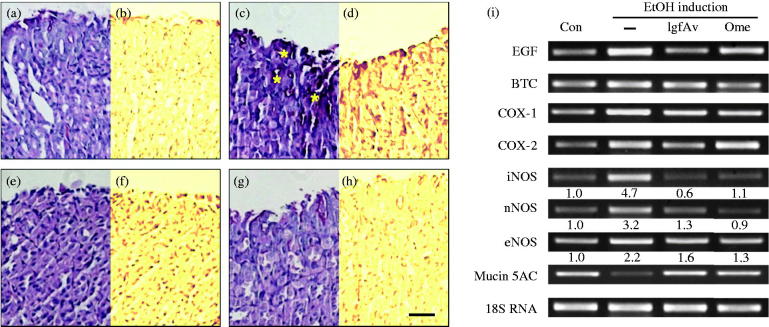
Immunohistochemical and reverse transcription-polymerase chain reaction (RT-PCR) analyses confirming inhibition by *Aloe vera* gel fraction. After deparaffinization, tissue sections were rehydrized and oxidized in 1% (v/v) periodic acid for 5 min. Then, sections were washed with distilled water (DW) and stained with Schiff′s reagent for 15 min. Thereafter, sections were counter-stained with Mayer′s haematoxylin and rinsed in running tap water. They were finally dehydrated, cleared and mounted. Sections of (a and b) control, (c and d), only alcohol-treated, (e and f) alcohol-treated with gel fraction and (g and h) omeprazole-treated groups were observed using phase contrast microscope (Axovert 200, Carl Zeiss, Weimar, Germany); (i) *Acute gastric lesions. Comparison of mRNA expressions of alcohol-induced acute lesions using RT-PCR. PCR conditions are described in Materials and Methods section. Data show representative classical analysis in triplicate independently.

We further confirmed that the tissue samples from the lgfAv-treated group showed stronger immunopositive activity than those from the alcohol- or omeprazole-treated groups did. As shown in Figure 3, the damage-induced scaring indicated that numerous inflammatory lesions were smeared by the PAS dye and turned extremely red, that is indicative of immunopositive cells. In contrast, the lgfAv-treated mouse tissues showed a more significant mitigation of the ulcerative pits and inflammatory infiltrates than the group treated with alcohol alone did (compare Figure 3(c,e)), indicating that the constituents of lgfAv relieved the inflammatory ulcer lesions. In addition, the intensity analysis of the captured images of the lesions also showed the same index as the haematoxylin and eosin (H&E) dye (Figure 3(b,d,f,h)). We further investigated the possible underlying molecular mechanisms of the effects against the alcohol-induced lesions in mice. We performed reverse transcription-polymerase chain reaction (RT-PCR) analysis to determine the transcriptional level of several markers related to inflammation during the gastric injury in mice treated with or without lgfAv. As shown in Figure 3(i), the expression of BTC, COX-1 and COX-2 during alcohol induction was not notably altered (columns 2–4 in Figure 3(i)). However, the mRNA level of EGF in the lgfAv-treated mice was slightly decreased more than that of the omeprazole (column 1 in Figure 3(i)). MUC5AC, an anti-inflammatory mediator, which is associated with mucin secretion, decreased when the stomach tissues were exposed to alcohol induction, while the expression levels apparently recovered following treatment with lgfAv (column 8 in Figure 3(i), compare column 2 to lanes 3 and 4). Very clearly, the other sensitive parameters iNOS and nNOS, not eNOS, observed altered expression pattern in lgfAv- or omeprazole-administered mice exposed to alcohol. These two isoforms may be associated with the exact mechanism for protection of the fraction (Figure 3, columns 5–7).

### MMP-9 protein expression is associated with lgfAv-induced gastroprotection against alcohol-induced gastric lesions

**Figure 4.**
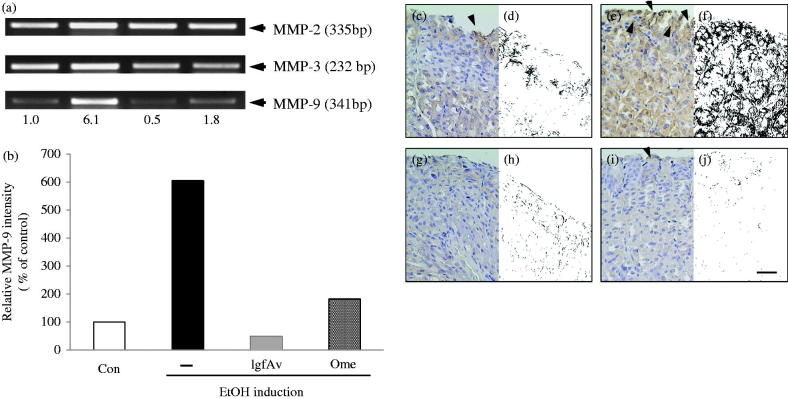
Matrix metalloproteinase (MMP)-9 protein expression is associated with gastroprotection by *Aloe vera* gel fraction against alcohol-induced acute gastric mucosal lesions. (a) mRNA expressions of MMP-2, -3 and -9 using reverse transcription-polymerase chain reaction (RT-PCR). Each tissue sample was homogenized, quantified and subjected to RT-PCR as described in the Materials and Methods section. (b) Relative band intensity is shown. (c, d) Sections of control, (e, f) only alcohol-treated, (g, h) alcohol-treated with lgfAv and (i, j) omeprazole-treated groups were immunohistochemically stained with MMP-9 antibody and visualized using secondary antibody. Dark blots (arrowheads) indicate MMP-9-responsive cells, which are secreted or bound with MMP-9 cells.

To prove the association of the MMPs in gastric inflammation, we determined the MMPs involved in the healing of the inflammation during the establishment of the alcohol-induced lesion model. As shown in Figure 4, the MMP-2 expression level was almost the same (Figure 4(a), column 1), but that of MMP-3 was slightly decreased (Figure 4(a), column 2). Surprisingly, MMP-9 showed a 6.2-fold higher activity in the expression than the control did (column 3 of Figure 4(a,b)). The activity was reduced by the lgfAv treatment (Figure 4(b), column 3), resulting in a decline to the control level, as shown by the control (Figure 4(b), compare to omeprazole group). As shown in Figure 4(c,e,g,i for ulcer index; d,f,h,j for image analysis) a dramatic decrease in the number of MMP-9-expressing cells was observed in the tissues from the alcohol-treated mice (arrowheads in Figure 4(e,f)). Furthermore, this effect was alleviated by lgfAv treatment (Figure 4(g,h)), whereas the omeprazole treated mice still exhibited damage-induced scars or clumps (arrowheads in Figure 4(i,j)).

## Discussion

In current study, we revealed that the lgfAv fraction decreased the alcohol-induced gastric lesions in a mouse model, and the underlying mechanism mainly involves inhibition of the mRNA expression of MMP-9. The MMP is a family of well-known proteases that are involved in tissue remodelling and the wound healing process (Ganguly and Swarnakar 2009; Rohani and Parks 2015; Latifa et al. 2016). The present results revealed that the expression pattern of MMP-9 depended on the intensity of the alcohol-induced gastric lesions (Figure 4). We could not obtain any significant differences in the MMP-2, -3 (Figure 4(a)) and -7 levels (data not shown). However, the MMP-9 mRNA expression levels were coarsely 6.2-fold stronger than that of the control was (Figure 4(b)). The MMP-9 expression was considerably reduced by lgfAv, and reached comparable levels as that of the control. By comparing the immunohistochemistry of the MMP-9 antibody, we found that the enzyme expression pattern was very similar to the expression intensity of the RT-PCR (Figure 4(b)), gastric lesions (Figure 2(a–h)), lesion index (Figure 2(i)) and the mRNA expression profile (Figure 3(i)).

This study has strong relevance because a mass scale production method for Av products has been developed for the preparation of each fraction of the gel ingredients in the leaves. We purified the gel fraction using ultrafiltration and by adjusting the MWCO to <50 kDa, which is the fraction that exhibited gastroprotective activity (data not shown). The characteristics of pulverized gel fraction showed light yellowish colour, not sticky, whereas those of polymer fraction (<50 kDa) did dark yellowish colour, a little sticky, and glossy, suggesting that the two samples are different from physiochemical properties (data not shown). This fraction has no acid-neutralizing capacity (Figure 1(b)) and, therefore, we hypothesized that the molecular mechanisms of the protection against alcohol-induced gastritis are associated with its wound healing properties in the tissues. In a previous report, we studied the polymer fraction of Av and discovered that it significantly protected against the development of gastric lesions associated with ethanol-induced ulcers. To further ascertain the molecular mechanisms of the other fractions, we investigated whether the low molecular-weight gel fraction exhibits gastroprotection in alcohol-induced acute tissues.

We first investigated the protective effects of lgfAv, which appeared to involve the inhibition of nitric oxide (NO) levels by way of downregulation of the mRNA expression of inducible NO synthase (iNOS). Previously, it is reported that the WIRS-induced gastric lesions were related to upregulated augmented gastric peristalsis, declined prostaglandin levels, disrupted microcirculation, and changed NO levels in the gastric mucosa cells (Izgüt-Uysal et al. 2014). The NO-facilitated regulation takes part in gastro-protection against injury induced by exogenous stresses such as drugs, and the mechanism involves NOS activity in the gastric tissues (Backer and Lefebvre 2008). As earlier mentioned, we confirmed the dramatic decrease in the expression of iNOS levels and neuronal NOS (nNOS) by lgfAv treatment, but the intensity was weaker than that of MMP-9 was. Therefore, we evaluated the expression levels of the MMPs.

Image analysis is a useful tool for the determination of the phenotypic symptoms, which uses photographic data, including immunohistochemical images, and computer software, such as ImageJ, Adobe Photoshop and Metamorph (Prasad and Prabhu 2012). This technique was applied to the photographic data (white and black photos in Figures 2–4), which enabled clear observation of the differences because the process removed unnecessary background information. Therefore, we were able to confirm that the specific areas of damaged tissues can be simultaneously evaluated by binary viewing of the lesion index and image analysis.

Here, we showed that the administration of lgfAv provided gastroprotection against the alcohol-induced gastric lesions, and this protective effect may be attributable to its mitigation of MMP-9 mRNA and protein expressions. Although future studies will concentrate on the molecular weight and structure determination of lgfAv and to elucidate its protective effect, these present findings provide a new insight for the nutraceutical and cosmeceutical use of *Aloe*. Furthermore, our results strongly suggest that the *Aloe* fractions could be developed for future inner beauty-purpose functional food ingredients as well as gastric preventive and therapeutic applications.

## Conclusions

*Aloe vera,* which is found worldwide, has been extensively investigated to improve its use as an anti-inflammatory or wound healing agent. As lgfAv showed no acid neutralizing capacity, we investigated the antiulcer mechanism of lgfAv and the biomarkers that were associated with gastroprotection. Here, we demonstrated that lgfAv in mucosal tissues had the potential to inhibit the mRNA and protein expression levels of MMPs and NOSs by using RT-PCR and immunohistochemistry, respectively. The protein expression of MMP-9 was closely associated with lgfAv-induced gastroprotection against alcohol-induced gastric lesions. The current results strongly suggested that this fraction of the *Aloe* gel could be developed into beautifying foods and biomaterials, as well as have future gastric prevention and therapeutic applications.
